# Increased Risk of Chronic Periodontitis in Chronic Rhinosinusitis Patients: A Longitudinal Follow-Up Study Using a National Health-Screening Cohort

**DOI:** 10.3390/jcm9041170

**Published:** 2020-04-19

**Authors:** Soo Hwan Byun, Chanyang Min, Il Seok Park, Heejin Kim, Sung Kyun Kim, Bum Jung Park, Hyo Geun Choi, Seok Jin Hong

**Affiliations:** 1 Department of Oral & Maxillofacial Surgery, Dentistry, Sacred Heart Hospital, Hallym University College of Medicine, Anyang 14068, Korea; purheit@daum.net; 2 Hallym Data Science Laboratory, Hallym University College of Medicine, Anyang 14068, Korea; joicemin@naver.com; 3 Graduate School of Public Health, Seoul National University, Seoul 03080, Korea; 4 Department of Otorhinolaryngology-Head & Neck Surgery, Dongtan Sacred Heart Hospital, Hallym University College of Medicine, Dongtan 18450, Korea; ispark@hallym.or.kr (I.S.P.); mir5020@hallym.or.kr (H.K.); newearera@hallym.or.kr (S.K.K.); 5 Department of Otorhinolaryngology-Head & Neck Surgery, Sacred Heart Hospital, Hallym University College of Medicine, Anyang 14068, Korea; bumjung426@gmail.com

**Keywords:** rhinosinusitis, rhinitis, periodontitis, inflammation, microbiota, bacteria, cohort, korean national health screening, systemic diseases

## Abstract

This study compared the risk of chronic periodontitis (CP) between chronic rhinosinusitis (CRS) and non-chronic rhinosinusitis (control) patients using a national cohort dataset from the Korean Health Insurance Review and Assessment Service. CRS (n = 5951) and control participants (n = 23,804) were selected after 1:4 ratio matching for age, sex, income, region of residence, and preoperative CP visits. Postoperative CP visits were measured between 2002 and 2015. The margin of equivalence of the difference between the CRS and control groups was set between −0.5 and 0.5. Statistical significance was noted in the post-index date (ID) of the third, fourth, and fifth year periods. In subgroup analyses according to age and sex, statistical significance was observed in 40–59-year-old males in post-ID third, fourth, and fifth year periods, ≥60-year-old males in post-ID third and fourth year periods, and ≥60-year-old females in post-ID fifth year period (*p* < 0.05, each). In another subgroup analysis based on the number of pre-ID CP visits, statistical significance was observed for pre-ID CP (0 time) in the third, fourth, and fifth year periods (*p* < 0.05, each). This study revealed that CRS participants were likely to receive CP diagnosis and treatment.

## 1. Introduction

Chronic rhinosinusitis (CRS) is one of the most prevalent diseases of the upper respiratory tract. The prevalence of adult CRS in the Korean population was 8.4% in a study analyzing five-year cross-sectional data from the Korean National Health and Nutrition Examination Survey [1]. CRS is one of the most prevalent chronic conditions in the United States, and the prevalence of adult CRS is 12.5% in the US population [2]. European position paper on rhinosinusitis (EPOS) 2012 defined CRS as an inflammation of the nose and paranasal sinuses characterized by two or more symptoms, one of which should be either nasal blockage/obstruction/congestion or nasal discharge (anterior/posterior nasal drip): ± facial pain/pressure ± reduction or loss of sense of smell [3].

Although the pathogenesis of CRS has not been clarified, the hypothesis is that the etiology could be related to abnormalities in the epithelial barrier function and mucociliary clearance, bacterial biofilms, tissue remodeling, the host innate and adaptive immune system, and microbiome dysbiosis [4]. CRS is considered a chronic inflammatory disease rather than infection, wherein commensal resident microbiota and pathogenic microbiota could play a crucial role in the initiation and progression of the mucosal inflammation [5,6].

Chronic periodontitis (CP) is an inflammatory disease of the gingiva, accompanied by the loss of supportive connective tissues, including the periodontal ligament and alveolar bone [7]. Worsening of periodontitis results in tooth mobility and loss. CP is highly prevalent, affecting about 35% of adults >30 years of age [8]. Periodontal disease is a common disease affecting approximately 47.2% (or 64.7 million) of the US adult population aged ≥30 years [9]. The pathogenesis of periodontitis involves complex interactions between bacteria, genetic factors, and environmental factors. Bacteria can initiate inflammatory reactions through interactions between pathogen-associated molecular patterns and pattern recognition receptors [10]. The most prevalent anaerobic Gram-negative bacteria involved in periodontitis are *Actinobacillus actinomycetemcomitans, Porphyromonas gingivalis, Prevotella intermedia*, and *Tannerella forsythensis* [11]. These bacteria play an important role in the onset and progression of periodontitis, formation of periodontal pockets, connective tissue destruction, and alveolar bone loss through immunologic and pathogenic mechanisms. Once periodontitis has been initiated, an inflammatory factor produces various cytokine subtypes and biological elements responsible for immunologic and pathogenic reactions. Microorganisms usually proliferate on dental surfaces in the form of plaque. A plaque is an agglomeration of biofilm, which is known to be a phenotype of bacteria. The spread of biofilms is of great clinical importance in periodontitis, since it is resistant to antimicrobial agents [12,13].

CRS and CP exhibit some common characteristics. Both are chronic conditions, and the polymicrobial biofilms are stabilized in the airway of patients with CRS or in the oral cavity of patients with CP. Previous large population studies have attempted to explain the association between CRS and CP by taking into account these similar characteristics [14,15].

The purpose of this study was to compare the risk of CP between the chronic rhinosinusitis (CRS group) and non-chronic rhinosinusitis (control group) participants using a national cohort dataset. In this study, the CRS and control groups were matched at a 1:4 ratio by adjusting for age, sex, region of residence, pre-index CP treatment, obesity, smoking, alcohol consumption, and Charlson comorbidity index (CCI) score.

## 2. Materials and Methods

### 2.1. Study Population

The study was approved by the ethics committee of Hallym University (2017-I102) and written informed consent was waived by the Institutional Review Board. All analyses adhered to the guidelines and regulations of the ethics committee of Hallym University. A detailed description of the Korean National Health Insurance Service-Health Screening Cohort data has been described previously [16].

### 2.2. Chronic Rhinosinusitis

CRS was defined using the 10th International Statistical Classification of Diseases and Related Health Problems (ICD-10) codes (J32). We selected participants who were treated ≥2 times, and those who underwent head and neck computed tomography evaluations (Claim codes: HA401-HA416, HA441-HA443, HA451-HA453, HA461-HA463, or HA471-HA473). Among the CRS patients, 4423 were treated for nasal polyps (J33), while the other 4137 participants were not.

### 2.3. Chronic Periodontitis

CP was defined based on the ICD-10 codes (K05.3) and treated by dentists. The number of CP treatments was counted from the date of CRS treatment (index date [ID]) to the date before the 2-year period (pre-ID CP for 2 y). The number of CP treatments was counted for periods from the ID to the date up to the end of the first year (post-ID 1 y CP, postoperative 1–365 days), second year (post-ID 2 y CP, postoperative 366–730 days), third year (post-ID 3 y CP, postoperative 731–1095 days), fourth year (post-ID 4 y CP, postoperative 1,096–1,460 days), and fifth year (post-ID 5 y CP, postoperative 1461–1825 days).

### 2.4. Participant Selection

**Figure 1:**
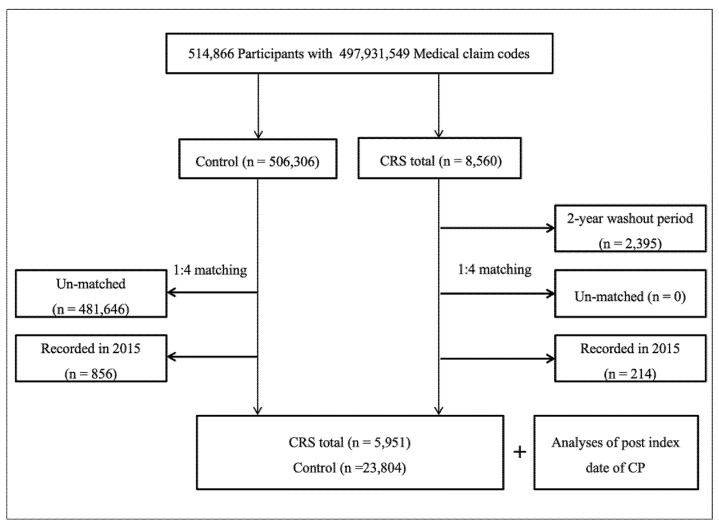
Schematic illustration of the participant selection process used in the present study. Out of 514,866 participants, 5951 chronic rhinosinusitis patients were matched with 23,804 control participants for age, sex, income, region of residence, and pre-index date chronic periodontitis for 2 years.

CRS patients were selected from 514,866 participants with 497,931,549 medical claim codes (n = 8560). The control group included participants who did not have CRS from 2002 to 2015 (n = 506,306). To select CRS patients who were diagnosed for the first time, we excluded CRS patients diagnosed between 2002 and 2003 (washout periods, n = 2395). CRS patients were matched at a 1:4 ratio with control group participants for age, sex, income, and region of residence. To analyze subgroups according to pre-ID CP for 2 y, CRS patients were additionally matched with pre-ID CP for 2 y with categorical variables (0 time, 1 time, and ≥2 times). To minimize selection bias, the control participants were randomly selected. The ID of each CRS patient was set as the date of their CRS treatment. The ID of the control participants was set as that of their matched CRS patients. Therefore, each CRS patient matched with a control participant had the same ID as the latter. During the 1:4 matching procedure, 481,646 un-matched control participants were excluded. Participants recorded in 2015 were excluded to calculate post-ID 1 y CP (n = 214 for CRS patients, n = 856 for control participants). Finally, 5951 CRS patients with or without nasal polyps were matched (1:4 ratio) with 23,804 control participants (Figure 1).

### 2.5. Other Variables

Age groups were divided into 5-year intervals, and ten age groups (40–44, 45–49, 50–54…, and 85+ years) were specified. Income groups were classified into five classes (class 1: lowest income to class 5: highest income). The regions of residence were grouped into urban (Seoul, Busan, Daegu, Incheon, Gwangju, Daejeon, and Ulsan) and rural (Gyeonggi, Gangwon, Chungcheongbuk, Chungcheongnam, Jeollabuk, Jeollanam, Gyeongsangbuk, Gyeongsangnam, and Jeju) areas.

Tobacco smoking was categorized based on the participant’s current smoking status (non-smoker, past smoker, and current smoker). Alcohol consumption was categorized based on the frequency of alcohol consumption (<1 time a week and ≥1 time a week). Obesity was measured using body mass index (BMI, kg/m^2^). Missing BMI variables were replaced by the mean BMI of the final selected participants. BMI was categorized as <18.5 (underweight), ≥18.5 to ≤23 (normal), ≥23 to <25 (overweight), ≥25 to <30 (obese I), and ≥30 (obese II) based on the Asia-Pacific criteria following the Western Pacific Regional Office 2000.

The CCI has been widely used to measure disease burden using 17 comorbidities. A score was given to each participant depending on the severity and number of diseases. CCI was measured as a continuous variable (0 (no comorbidities) through 29 (multiple comorbidities)) [17,18]. The scores were calculated after the exclusion of cerebrovascular diseases. The CCI score was used as a covariate in the analyses.

### 2.6. Statistical Analyses

The general characteristics between the CRS and control groups were compared using the chi-square test. Simple and multiple linear regressions were used to calculate estimated values and 95% confidence intervals (CI) for post-ID 1 y CP, post-ID 2 y CP, post-ID 3 y CP, post-ID 4 y CP, and post-ID 5 y CP, in CRS patients with or without nasal polyps, and compared to the control group. Simple and multiple linear regressions were stratified by age, sex, income, and region of residence. In multiple linear regression, the model was adjusted for obesity, smoking status, alcohol consumption, CCI score, and pre-ID CP for 2 y with continuous variables.

For the subgroup analyses, we divided participants by age (<60 years old and ≥60 years old), sex (male and female), and pre-ID CP for 2 y (0 time, 1 time, and ≥2 times variables) and analyzed the crude and adjusted models.

Two-tailed analyses were performed, and significance was defined as a *p*-value <0.05. SAS version 9.4 (SAS Institute Inc., Cary, NC, USA) was used for statistical analyses.

## 3. Results

Age, sex, income, and region of residence were similar in the CRS and control groups (*p* = 1.000), while smoking and CCI scores were different between both groups (*p* < 0.05, Table 1).

The number of CP cases before the ID was matched as the categorical variable. The adjusted estimated value (EV) of the number of post-ID CP cases did not reach statistical significance in post-ID 1 y and 2 y (*p* > 0.05, each; Table 2).

However, it showed statistical significance for 3 y (EV = 0.071, 95% CI = 0.030–0.113), 4 y (EV = 0.085, 95% CI = 0.040–0.130), and 5 y (EV = 0.057, 95% CI = 0.009–0.106; *p* < 0.05, each)

In the subgroup analyses according to age and sex, statistical significance was seen in 40–59-year-old males in post-ID 3 y, 4 y, and 5 y periods, ≥60-year-old males in post-ID 3 y and 4 y periods, and ≥60-year-old females in post-ID 5 y period (*p* < 0.05, each; Table 3).

In another subgroup analysis according to the number of pre-ID CP cases, a statistical significance for pre-ID CP (0 time) in post ID 3 y, 4 y, and 5 y was found (*p* < 0.05, each; Table 4).

## 4. Discussion

CRS and CP are associated with chronic inflammation. As CP is a widespread immunoinflammatory condition, an association between CRS and CP has been proposed. This was mainly attributed to increased levels of pro-inflammatory mediators, such as interleukin (IL)-1, IL-6, and tumor necrosis factor-α in the plasma [19]. Therefore, we sought to investigate the association between CRS and CP in a large population cohort.

This study revealed an increased risk for CP in post-ID three-, four-, and five-year periods following a diagnosis of CRS. In another subgroup analysis according to the number of pre-ID CP check-ups, statistical significance was observed for pre-ID CP (0 time) in post-ID three-, four-, and five-year periods. After adjusting for age, sex, income, region of residence, pre-operative CP, obesity, smoking, alcohol consumption, and CCI score, the study showed that participants with CRS were more likely to receive a CP diagnosis and treatment.

Various studies have demonstrated a relationship between mouth breathing and gingivitis in teenagers [20]. We can assume that CRS may induce mouth breathing in adults. Mouth breathing is related to dry mouth, which reduces the washing out of bacteria and biofilm by salivation. Chronic mouth breathing habits cause gingival inflammation, and further progression could develop CP due to biofilm and microbiome dysbiosis. This mechanism could be one of the possible explanations for the increased risk of CP with CRS in the present study. In particular, the adjusted estimated value of the number of post-ID CP cases did not reach statistical significance one and two years post-ID. Instead, it showed statistical significance three-, four-, and five-year post-ID. These results can be explained by the fact that it would take some time for CRS to cause mouth breathing, thereby affecting the periodontal tissue.

A recent study of CRS and CP suggested that a dysbiotic biofilm elicited inflammatory sinusitis and oral diseases in the host [21,22]. This theory states that biofilm agglomeration stabilizes a microbial profile that destroys equilibrium in the host [23]. This microbiome dysbiosis theory hypothesized that *Staphylococcus aureus, Streptococcus, Pseudomonas,* and *Anaerobes* play key roles as crucial pathogens in CRS. *Porpyromonas gingivalis* acts as an essential pathogen in CP, which can increase the activity of biofilm bacteria by interrupting homeostasis in the host [5,23,24]. Microbiome dysbiosis disrupts homeostasis between the resident microbiota and the host immune system, causing chronic inflammation that induces CRS and CP [5,25].

Another explanation would be the immunological response to environmental factors [26,27]. The accelerated immune response to the microbiota could be related to both CRS and CP. In the respiratory epithelium of CRS patients and in the oral epithelium of CP patients, polymicrobial biofilms were colonized, and the biofilms were proven to be a signal source for both innate and adaptive mucosal immune responses [28,29]. Chronic exposure to pathogens, such as bacteria, viruses, fungal spores, and environmental stressors, can cause respiratory epithelial cells and oral epithelial cells to secrete cytokines (IL-1, IL-6, IL-17, TNF-α, etc.). These cytokines activate inflammatory pathways and recruit dedicated immune cells (macrophages, dendritic cells, eosinophils, neutrophils, T cells, and NK cells) that could play a significant role in CRS and CP pathogenesis [4,30]. Microbiome dysbiosis, biofilms, and the polarization of cytokine patterns are essential for understanding the link between CRS and CP.

There are five major strengths of our study. The first advantage of this study is the large number of study participants (n = 29,755). The CRS group was followed-up for a maximum of 13 years, whereas another study conducted a five-year follow-up [14]. Second, the Korean National Health Insurance Service-Health Screening Cohort data was a large national survey that is representative of the Korean population. These cohort records were available for each participant. Previous studies asked participants about their history of disease, which could result in a recall bias [31,32]. On the other hand, the records used in our study were not distorted by the patient’s memory. The data also involved only Koreans, and all participants without exception. Therefore, no participants were missed during the follow-up period. Third, well-trained clinicians documented the general health examinations and laboratory evaluations. Fourth, professional dentists conducted periodontal diagnosis and treatment. These results support the evidence of an association between CP and CRS in adults. Finally, adjusting factors showed a statistically significant independent association with CRS in our data, thus confirming the reliability of our study.

The associations found in this study were obtained by using large population data. Nevertheless, the findings had limitations. First, although the dataset included many factors such as obesity, smoking, alcohol consumption, and age, it was impossible to adjust for all systemic factors such as sugar consumption, personal oral hygiene, and oral drugs, which were not included in the large population dataset in this study. Second, this study may have presented a surveillance bias. Patients with CRS were more susceptible to be diagnosed and treated for possibly unrelated CP based on their frequent visits to the medical institutions. However, it is very unlikely that increased patient visits would induce the detection of CP. Dental examinations were performed at an annual check-up visit covered by the Korean National Health Service (KNHS), which has exclusive characteristics including widespread coverage, efficient benefits, low payment, and easy access to medical institutions in Korea. In addition to these advantages, most Koreans have undergone regular dental check-ups without discomfort. Furthermore, the large population data of this study were adjusted for many factors, thereby minimizing the surveillance bias. Therefore, this study has probably avoided or minimized surveillance bias by adjusting the characteristics of the KNHS system.

## 5. Conclusions

This study revealed that CRS patients were more likely to receive CP diagnosis and treatment. CRS and CP are linked to chronic inflammation. Clinicians should be aware of the possible risk of CP in patients with CRS.

## Figures and Tables

**Table 1:** General Characteristics of Participants.

Characteristics	Total Participants		
	CRS Total (n, %)	Control (n, %)	*p*-Value
Age (years old)			1.000
40–44	233 (3.9)	932 (3.9)	
45–49	972 (16.3)	3888 (16.3)	
50–54	1323 (22.2)	5292 (22.2)	
55–59	1245 (20.9)	4980 (20.9)	
60–64	934 (15.7)	3736 (15.7)	
65–69	671 (11.3)	2684 (11.3)	
70–74	363 (6.1)	1452 (6.1)	
75–79	159 (2.7)	636 (2.7)	
80–84	40 (0.7)	160 (0.7)	
85+	11 (0.2)	44 (0.2)	
Sex			1.000
Male	3650 (61.3)	14,600 (61.3)	
Female	2301 (38.7)	9204 (38.7)	
Income			1.000
1 (lowest)	720 (12.1)	2880 (12.1)	
2	708 (11.9)	2832 (11.9)	
3	905 (15.2)	3620 (15.2)	
4	1294 (21.7)	5176 (21.7)	
5 (highest)	2324 (39.1)	9296 (39.1)	
Region of residence			1.000
Urban	2762 (46.4)	11,048 (46.4)	
Rural	3189 (53.6)	12,756 (53.6)	
Pre index date of CP			1.000
0 time	3818 (64.2)	15,272 (64.2)	
1 time	895 (15.0)	3580 (15.0)	
≥2 times	1238 (20.8)	4952 (20.8)	
Obesity ^†^			0.183
Underweight	101 (1.7)	445 (1.9)	
Normal	1933 (32.5)	8024 (33.7)	
Overweight	1780 (29.9)	6783 (28.5)	
Obese I	1973 (33.2)	7897 (33.2)	
Obese II	164 (2.8)	655 (2.8)	
Smoking status			0.004 *
Nonsmoker	3918 (65.8)	15,582 (65.5)	
Past smoker	858 (14.4)	3142 (13.2)	
Current smoker	1175 (19.7)	5080 (21.3)	
Alcohol consumption			0.858
<1 time a week	3972 (66.8)	15,917 (66.9)	
≥1 time a week	1979 (33.3)	7887 (33.1)	
CCI score			<0.001 *
0	3842 (64.6)	16,956 (71.2)	
1	965 (16.2)	3173 (13.3)	
2	544 (9.1)	1683 (7.1)	
3	263 (4.4)	841 (3.5)	
≥4	337 (5.7)	1151 (4.8)	
CRS total	5951 (100.0)	0 (0.0)	<0.001 *
CRS with nasal polyp	2891 (100.0)	0 (0.0)	<0.001 *
CRS without nasal polyp	3060 (100.0)	0 (0.0)	<0.001 *

Abbreviations: CCI, Charlson comorbidity index; CP, chronic periodontitis; CRS, Chronic Rhinosinusitis. * χ^2^ test. Significance at *p* < 0.05. ^†^ Obesity (BMI, body mass index, kg/m^2^) was categorized as <18.5 (underweight), ≥18.5 to <23 (normal), ≥23 to <25 (overweight), ≥25 to <30 (obese I), and ≥30 (obese II).

**Table 2:** Simple and multiple linear regression model (estimated value (95% confidence interval)) for post index date of CP (post-ID CP) periods in CRS with/without nasal polyp (total) group compared to control group.

Characteristics	Linear Regression			
	Simple ^†^	*p*-Value	Multiple ^†^^‡^	*p*-Value
Post ID 1 y CP (n = 29,755)				
CRS total	0.015 (−0.021 to 0.051)	0.417	0.005 (−0.029 to 0.040)	0.763
Post ID 2 y CP (n = 28,530)				
CRS total	0.004 (−0.035 to 0.043)	0.841	−0.003 (−0.041 to 0.035)	0.867
Post ID 3 y CP (n = 26,820)				
CRS total	0.078 (0.035 to 0.120)	<0.001 *	0.071 (0.030 to 0.113)	0.001 *
Post ID 4 y CP (n = 25,000)				
CRS total	0.087 (0.041 to 0.132)	<0.001 *	0.085 (0.040 to 0.130)	<0.001 *
Post ID 5 y CP (n = 22,660)				
CRS total	0.060 (0.011 to 0.109)	0.017 *	0.057 (0.009 to 0.106)	0.020 *

Abbreviations: CCI, Charlson comorbidity index; CP, chronic periodontitis; CRS, Chronic Rhinosinusitis. * Linear regression model, Significance at *p* < 0.05. ^†^ Models stratified by age, sex, income, and region of residence. ^‡^ A model adjusted for obesity, smoking, alcohol consumption, CCI scores, and pre index date of CP (pre-ID CP) for 2y.

**Table 3:** Subgroup analyses of simple and multiple linear regression model (estimated value (95% confidence interval)) for post index date of CP (post-ID CP) periods in CRS with/without nasal polyp group compared to control group according to age and sex.

	Linear Regression			
	Simple ^†^	*p*-Value	Multiple ^†^^‡^	*p*-Value
Age 40–59 years old males				
Post-ID 1 y CP (n = 12,035, Mean ± SD = 0.46 ± 1.19 for CRS, Mean ± SD = 0.48 ± 1.31 for control)				
CRS total	−0.020 (−0.077 to 0.037)	0.496	−0.037 (−0.093 to 0.018)	0.186
Post-ID 2 y CP (n = 11,635, Mean ± SD = 0.54 ± 1.37 for CRS, Mean ± SD = 0.53 ± 1.43 for control)				
CRS total	0.009 (−0.055 to 0.073)	0.777	−0.003 (−0.066 to 0.060)	0.933
Post-ID 3 y CP (n = 11,180, Mean ± SD = 0.63 ± 1.56 for CRS, Mean ± SD = 0.54 ± 1.40 for control)				
CRS total	0.084 (0.017 to 0.150)	0.013*	0.074 (0.009 to 0.139)	0.027 *
Post-ID 4 y CP (n = 10,565, Mean ± SD = 0.70 ± 1.62 for CRS, Mean ± SD = 0.61 ± 1.53 for control)				
CRS total	0.097 (0.023 to 0.170)	0.010*	0.090 (0.017 to 0.163)	0.015 *
Post-ID 5 y CP (n = 9705, Mean ± SD = 0.70 ± 1.60 for CRS, Mean ± SD = 0.65 ± 1.55 for control)				
CRS total	0.055 (−0.022 to 0.133)	0.164	0.044 (−0.033 to 0.121)	0.263
Age 40–59 years old females				
Post-ID 1 y CP (n = 6830, Mean ± SD = 0.44 ± 1.30 for CRS, Mean ± SD = 0.37 ± 1.07 for control)				
CRS total	0.065 (−0.001 to 0.131)	0.054	0.065 (0.002 to 0.129)	0.043 *
Post-ID 2 y CP (n = 6690, Mean ± SD = 0.42 ± 1.11 for CRS, Mean ± SD = 0.41 ± 1.20 for control)				
CRS total	0.010 (−0.061 to 0.080)	0.788	0.008 (−0.061 to 0.078)	0.817
Post-ID 3 y CP (n = 6420, Mean ± SD = 0.50 ± 1.25 for CRS, Mean ± SD = 0.47 ± 1.32 for control)				
CRS total	0.030 (−0.050 to 0.109)	0.467	0.031 (−0.048 to 0.110)	0.441
Post-ID 4 y CP (n = 6095, Mean ± SD = 0.47 ± 1.20 for CRS, Mean ± SD = 0.49 ± 1.31 for control)				
CRS total	−0.018 (−0.098 to 0.063)	0.669	−0.015 (−0.095 to 0.064)	0.705
Post-ID 5 y CP (n = 5615, Mean ± SD = 0.55 ± 1.21 for CRS, Mean ± SD = 0.54 ± 1.40 for control)				
CRS total	0.006 (−0.082 to 0.095)	0.887	0.012 (−0.076 to 0.100)	0.790
Age ≥60 years old males				
Post-ID 1 y CP (n = 6215, Mean ± SD = 0.65 ± 1.56 for CRS, Mean ± SD = 0.57 ± 1.39 for control)				
CRS total	0.083 (−0.005 to 0.172)	0.064	0.079 (−0.005 to 0.163)	0.066
Post-ID 2 y CP (n = 5795, Mean ± SD = 0.64 ± 1.37 for CRS, Mean ± SD = 0.62 ± 1.49 for control)				
CRS total	0.013 (−0.082 to 0.107)	0.795	0.006 (−0.086 to 0.097)	0.900
Post-ID 3 y CP (n = 5225, Mean ± SD = 0.80 ± 1.74 for CRS, Mean ± SD = 0.63 ± 1.51 for control)				
CRS total	0.172 (0.066 to 0.277)	0.002 *	0.165 (0.062 to 0.268)	0.002 *
Post-ID 4 y CP (n = 4670, Mean ± SD = 0.81 ± 1.86 for CRS, Mean ± SD = 0.61 ± 1.52 for control)				
CRS total	0.198 (0.085 to 0.312)	0.001 *	0.195 (0.083 to 0.307)	0.001 *
Post-ID 5 y CP (n = 4030, Mean ± SD = 0.77 ± 1.83 for CRS, Mean ± SD = 0.68 ± 1.68 for control)				
CRS total	0.086 (−0.046 to 0.217)	0.203	0.088 (−0.041 to 0.218)	0.182
Age ≥60 years old females				
Post-ID 1 y CP (n = 4675, Mean ± SD = 0.42 ± 1.13 for CRS, Mean ± SD = 0.48 ± 1.23 for control)				
CRS total	−0.060 (−0.147 to 0.027)	0.175	−0.066 (−0.150 to 0.017)	0.120
Post-ID 2 y CP (n = 4410, Mean ± SD = 0.43 ± 1.08 for CRS, Mean ± SD = 0.46 ± 1.28 for control)				
CRS total	−0.030 (−0.121 to 0.062)	0.523	−0.033 (−0.122 to 0.056)	0.464
Post-ID 3 y CP (n = 3995, Mean ± SD = 0.51 ± 1.59 for CRS, Mean ± SD = 0.49 ± 1.28 for control)				
CRS total	0.015 (−0.089 to 0.119)	0.773	0.009 (−0.093 to 0.112)	0.856
Post-ID 4 y CP (n = 3670, Mean ± SD = 0.57 ± 1.58 for CRS, Mean ± SD = 0.49 ± 1.28 for control)				
CRS total	0.089 (−0.020 to 0.198)	0.109	0.089 (−0.019 to 0.196)	0.106
Post-ID 5 y CP (n = 3310, Mean ± SD = 0.63 ± 1.53 for CRS, Mean ± SD = 0.50 ± 1.25 for control)				
CRS total	0.131 (0.019 to 0.243)	0.022 *	0.136 (0.025 to 0.247)	0.017 *

Abbreviations: CCI, Charlson comorbidity index; CP, chronic periodontitis; CRS, Chronic Rhinosinusitis. * Linear regression model, Significance at *p* < 0.05. ^†^ Models stratified by age, sex, income, and region of residence. ^‡^ A model adjusted for obesity, smoking, alcohol consumption, CCI scores, and pre index date of CP (pre-ID CP) for 2y.

**Table 4:** Subgroup analyses of simple and multiple linear regression model (estimated value [95% confidence interval]) for post index date of CP (post-ID CP) periods in CRS with/without nasal polyp and control groups according to pre index date of CP (pre-ID CP) for 2 years.

Characteristics	Linear Regression			
	Simple ^†^	*p*-Value	Multiple ^†^^‡^	*p*-Value
Pre-ID CP = 0 time for 2 years				
Post-ID 1 y CP (n = 19,090, Mean ± SD = 0.29 ± 0.91 for CRS, Mean ± SD = 0.29 ± 0.95 for control)				
CRS total	0.005 (−0.029 to 0.038)	0.780	0.006 (−0.028 to 0.039)	0.746
Post-ID 2 y CP (n = 18,530, Mean ± SD = 0.36 ± 1.04 for CRS, Mean ± SD = 0.35 ± 1.11 for control)				
CRS total	0.013 (−0.027 to 0.052)	0.525	0.015 (−0.024 to 0.054)	0.455
Post-ID 3 y CP (n = 17,665, Mean ± SD = 0.45 ± 1.32 for CRS, Mean ± SD = 0.38 ± 1.13 for control)				
CRS total	0.064 (0.021 to 0.107)	0.004 *	0.065 (0.022 to 0.108)	0.003 *
Post-ID 4 y CP (n = 16,715, Mean ± SD = 0.52 ± 1.43 for CRS, Mean ± SD = 0.41 ± 1.22 for control)				
CRS total	0.108 (0.060 to 0.156)	<0.001 *	0.111 (0.064 to 0.159)	<0.001 *
Post-ID 5 y CP (n = 15,410, Mean ± SD = 0.52 ± 1.29 for CRS, Mean ± SD = 0.47 ± 1.26 for control)				
CRS total	0.050 (0.000 to 0.100)	0.050 *	0.052 (0.002 to 0.102)	0.040 *
Pre-ID CP = 1 time for 2 years				
Post-ID 1 y CP (n = 4475, Mean ± SD = 0.51 ± 1.19 for CRS, Mean ± SD = 0.54 ± 1.27 for control)				
CRS total	−0.030 (−0.121 to 0.062)	0.526	−0.029 (−0.120 to 0.063)	0.540
Post-ID 2 y CP (n = 4270, Mean ± SD = 0.55 ± 1.25 for CRS, Mean ± SD = 0.60 ± 1.40 for control)				
CRS total	−0.052 (−0.154 to 0.051)	0.321	−0.051 (−0.153 to 0.051)	0.329
Post-ID 3 y CP (n = 4005, Mean ± SD = 0.67 ± 1.54 for CRS, Mean ± SD = 0.62 ± 1.50 for control)				
CRS total	0.046 (−0.071 to 0.163)	0.438	0.053 (−0.063 to 0.170)	0.370
Post-ID 4 y CP (n = 3665, Mean ± SD = 0.76 ± 1.68 for CRS, Mean ± SD = 0.66 ± 1.52 for control)				
CRS total	0.095 (−0.031 to 0.220)	0.139	0.097 (−0.029 to 0.223)	0.130
Post-ID 5 y CP (n = 3265, Mean ± SD = 0.83 ± 1.90 for CRS, Mean ± SD = 0.71 ± 1.63 for control)				
CRS total	0.125 (−0.020 to 0.269)	0.090	0.128 (−0.017 to 0.272)	0.083
Pre-ID CP ≥ 2 times for 2 years				
Post-ID 1 y CP (n = 6190, Mean ± SD = 1.08 ± 2.02 for CRS, Mean ± SD = 1.00 ± 1.82 for control)				
CRS total	0.078 (−0.038 to 0.194)	0.186	0.031 (−0.081 to 0.143)	0.589
Post-ID 2 y CP (n = 5730, Mean ± SD = 1.00 ± 1.76 for CRS, Mean ± SD = 0.98 ± 1.90 for control)				
CRS total	0.017 (−0.104 to 0.138)	0.780	−0.023 (−0.142 to 0.097)	0.710
Post-ID 3 y CP (n = 5150, Mean ± SD = 1.14 ± 2.03 for CRS, Mean ± SD = 0.99 ± 1.88 for control)				
CRS total	0.148 (0.017 to 0.279)	0.027 *	0.122 (−0.007 to 0.251)	0.064
Post-ID 4 y CP (n = 4620, Mean ± SD = 1.02 ± 1.90 for CRS, Mean ± SD = 1.01 ± 1.94 for control)				
CRS total	0.003 (−0.137 to 0.142)	0.970	−0.013 (−0.152 to 0.126)	0.853
Post-ID 5 y CP (n = 3985, Mean ± SD = 1.09 ± 2.02 for CRS, Mean ± SD = 1.05 ± 2.04 for control)				
CRS total	0.043 (−0.115 to 0.201)	0.592	0.035 (−0.123 to 0.192)	0.667

Abbreviations: CCI, Charlson comorbidity index; CP, chronic periodontitis; CRS, Chronic Rhinosinusitis. * Linear regression model, Significance at *p* < 0.05. ^†^ Models stratified by age, sex, income, and region of residence. ^‡^ A model adjusted for obesity, smoking, alcohol consumption, CCI scores, and pre-ID CP for 2y.
